# Green Synthesis of Bacopa monnieri-Mediated Magnesium Oxide Nanoparticles and Analysis of Their Antimicrobial, Antioxidant, and Cytotoxic Properties

**DOI:** 10.7759/cureus.52701

**Published:** 2024-01-22

**Authors:** Yashwini Srinivasan, Parkavi Arumugam, Saheb Ali

**Affiliations:** 1 Periodontics, Saveetha Dental College and Hospitals, Saveetha Institute of Medical and Technical Sciences, Saveetha University, Chennai, IND

**Keywords:** environment, green synthesis, antioxidants, antimicrobials, nanoparticles, periodontal disease

## Abstract

Background

The management of aggressive forms of periodontal disease has become an issue of concern due to the emergence of bacterial resistance. Nanoparticles (NPs) have emerged as a potential therapeutic agent with a multitude of biological functions. The green synthesis of these NPs is more eco-friendly than conventional methods. The present study aimed at the green synthesis of magnesium oxide nanoparticles using *Bacopa monnieri* (bMgO NPs) and its antibacterial, antioxidant, and cytotoxic analysis.

Materials and methods

Magnesium oxide NPs were green synthesized using *B. monnieri* extract using a wet chemical method. The resultant bMgO NPs were assessed for antibacterial activity against *Staphylococcus aureus *and *Escherichia coli*. Antioxidant activity was assessed using the 1,1-diphenyl-2-picrylhydrazyl (DPPH) assay and the hydrogen peroxide (H_2_O_2_) assay. Cytotoxicity was assessed using zebrafish viability on treatment with bMgO NPs.

Results

Compared to the antibiotic standard, the green synthesized bMgO NPs showed good antibacterial properties against *S. aureus *and *E. coli*. It also showed excellent antioxidant activity and biocompatibility.

Conclusion

The bMgO NPs have great potential as a local drug delivery agent and should be further explored for their antibacterial and antioxidant properties in vivo.

## Introduction

Periodontal disease is one of the most common oral conditions affecting the global population. Approximately 1.1 billion individuals were affected by severe periodontal disease globally in 2019 [1]. An increase in the prevalence and severity of the disease in older populations is attributed to an increase in the presence of local factors, which is further corroborated by systemic influences. It is an inflammatory disease that initiates in response to microbial dental plaque. The periodontal bacteria, predominantly but not limited to the red complex bacteria like *Porphyromonas gingivalis*, *Treponema denticola*, and *Tannerella forsythia* in dental plaque, release an array of endotoxins like lipopolysaccharides, lipoteichoic acid, noxious enzymes, extracellular DNA, virulence factors, fimbriae, flagella, capsules, and proteins that result in an ensuing host response mediated by the release of cytokines, chemokines, and tissue-destructing agents. Interleukins, prostaglandins, prostacyclins, matrix metalloproteinases, and tumor necrosis factors play a significant role in the further propulsion of the disease process. This host-mediated response initiates the activation of the immune responses that direct the enrollment of neutrophils, lymphocytes, macrophages, plasma cells, B cells, and T cells, leading to bacterial killing. A predominance of B cells and plasma cells is evident in the established and advanced stages of periodontitis. The immuno-inflammatory response, although primarily protective, also causes a bystander effect with increased tissue destruction. The extent of the host response may be classified as a hypo-response, a normo-response, or a hyper-response, which is controlled by various factors like genetic, epigenetic, systemic, and environmental patterns. The resultant tissue breakdown products formed are in turn used by the microbial flora, causing a dysbiotic environment with the emergence of colonies of periopathogens.

Periodontal pathogenesis is not limited to the immune and inflammatory arms of the disease. It is also complicated by the production of reactive species during the process of microbial neutralization. Reactive species of the oxygen, nitrogen, and chlorine families, such as superoxide ion, hydrogen peroxide (H_2_O_2_), hydroxyl radical, nitroxyl ion, and chloride radical, produced during microbial killing, generate a chain reaction of free radicals that further propagates tissue destruction [2]. This leads to an imbalance between innate antioxidants and the generated reactive species, further aggravating the destructive process. Hence, a vicious propagative tissue-destructive cycle is established that can only be broken by a therapeutic rationale.

Various non-surgical and surgical therapies that aim at the complete removal of etiologic factors have been employed. However, it has been observed that a few periopathogens, namely *P. gingivalis*, *Aggregatibacter actinomycetemcomitans*, and *Fusobacterium nucleatum*, have the ability for intraepithelial colonization, where they act as nodes for pocket recolonization even following mechanical therapy [3]. Moreover, the injudicious use of systemic antibiotics has also led to the emergence of super-resistant bacterial species that have been causing aggressive forms of tissue destruction, ineffective responses to treatment, and fungal infections [4]. Also, systemic antibiotic use has been associated with several side effects like allergies, nausea, vomiting, altered taste sensation, ulcers, etc. All these factors have paved the way for newer therapeutic agents with combined antimicrobial and antioxidant properties that can be used as local drug delivery agents in the form of gels, microspheres, nanofibers, and mouthwashes. They can provide personalized treatment options with optimized concentration, targeted action, and minimal side effects.

Nanotechnology has emerged as a promising field with potential applications in the diagnosis and management of multiple conditions [5,6]. Nanomaterials are particles of matter ranging in nanometer scale, and they are available in different dimensions, such as nanoparticles (NPs), nanorods, nanosensors, and nanorobots. Metal oxide NPs have gained significant attention due to their bioavailability, increased surface-volume ratio, nanoscale size, reactivity, toughness, and optical properties. In contrast to traditional medicine, nanomedicine enables targeted drug delivery, reduced cytotoxicity, improved solubility, permeability, and bioavailability with prolonged release of drugs, making nanomedicine a more effective and safer therapeutic modality. These NPs have been explored for their potential antioxidant, antimicrobial, anti-inflammatory, and anti-carcinogenic activity [7,8]. Gold-, silver-, copper-, and zinc-based NPs have been commonly studied. However, very few studies have explored the potential of magnesium oxide nanoparticles (MgO NPs). They are extremely biocompatible, non-toxic, and highly stable metal oxide NPs with potential biological applications.

Conventionally, NPs are synthesized by different methods like mechanical milling, phyhsical and chemical vapor deposition, sol-gel, colloidal method, spray pyrolysis, sonochemical synthesis, hydrothermal synthesis, coprecipitation, etc. These traditional methods are associated with disadvantages like heat and toxic byproduct generation, the use of harsh chemicals, and costly equipment. They have grave negative effects on the ecosystem. Alternative green methods for the synthesis of NPs have been developed. Biologic sources like plants, herbs, microorganisms, and biomolecule-based extracts are used to synthesize NPs.

Various herbs and plants that are commonly available and used in our day-to-day lives, such as neem, tulasi, turmeric, and ginger, have been explored for green synthesis. We chose a herb that is native to our South Asian roots and has significant medicinal value but has not been commonly used for green synthesis. The present study used *Bacopa monnieri* extract to synthesize MgO NPs. *B. monnieri*, also known as Brahmi, water hyssop, or herb of grace, is commonly found on the South Asian continent. Over centuries, it has been employed to treat neurological diseases; improve memory, cognition, and learning; and reduce inflammation, anxiety, and stress. They are a rich source of phytochemicals like alkaloids, nicotine, bacosides A and B, flavonoids, tannins, saponins, and phytosterols, providing this herb with various potential biologic antimicrobial, anti-inflammatory, antioxidant, and anti-cancer properties [9]. Exploring their pharmaceutical potential would pave the way for identifying natural sources of potential drugs that can be applied in the treatment of various diseases, including periodontal disease. To the best of our knowledge, no other study has analyzed the biological properties of green synthesized magnesium oxide nanoparticles using *B. monnieri* (bMgO NPs). This study aimed to synthesize bMgO NPs and analyze their antimicrobial, antioxidant, and cytotoxic properties.

## Materials and methods

The study was conducted at the Department of Biomaterials at Saveetha Dental College in Chennai, Tamil Nadu, India. Approval was obtained from the Institutional Ethical Committee for Animal Research, Saveetha Dental College (Protocol number: BRULAC/SDCH/SIMATS/IAEC/06-2023/15) for the conduction of biocompatibility analysis using zebrafish embryos.

Sample collection

Fresh leaves of *B. monnieri* plants were obtained from Havelock Island, Andaman and Nicobar, India. Following the thorough cleaning of the collected leaves using distilled water, they were dried completely, powdered using a grinder, and stored at room temperature until further use.

Synthesis of MgO NPs

A wet chemical method was performed for the green synthesis of bMgO NPs using *B. monnieri*. A total of 15 g of *B. monnieri *leaf powder was mixed with 100 ml of distilled water. This mixture was agitated at a constant speed of 125 rpm at room temperature for 24 hours. The resultant plant extract was filtered, to which 10 ml of zinc acetate was added and heated until a white precipitate was obtained. The solution was washed with ethanol and centrifuged to collect the pellet.

Antibacterial activity

The green synthesized bMgO NPs were analyzed for their antibacterial properties against *Staphylococcus aureus *MTCC 2639 and *Escherichia coli* MTCC 1692. The bacteria were cultured on Mueller-Hinton agar plates. The bMgO NPs and antibiotic control loaded discs were placed on the agar plate. A combination of erythromycin 25 mg and amoxicillin 25 mg in a 1:1 ratio was used as the control. The bMgO NP was loaded in two different concentrations. Following incubation, the zone of inhibition of bacterial growth was assessed around the discs to analyze the antibacterial activity.

1,1-diphenyl-2-picrylhydrazyl (DPPH) assay

The radical-scavenging capability of bMgO NPs was evaluated using the DPPH assay method. The stock solution was prepared by mixing 22 mg of DPPH in 100 ml of methanol, which was filtered to obtain a compound with an absorbance of around 0.892 at 517 nm. DPPH and bMgO NPs were mixed in different ratios. The standard was 3 ml of DPPH solution in 100 µL of methanol. After 30 minutes, the solution was assessed for absorbance at 517 nm. The percentage of antioxidants was calculated and plotted.

H_2_O_2_ assay

The H_2_O_2_ scavenging activity of bMgO NPs was determined using the classical UV method at 230 nm. H_2_O_2_ scavenging activity was assessed using a calorimetric method. The reaction of H_2_O_2_ with phenol and 4-aminoantipyrine in the presence of horseradish peroxidase produces a pink quinoneimine dye. This color change was assessed to determine the scavenging activity. This calorimetric assay was applied to standard antioxidant ascorbic acid in addition to selected bMgO NPs in different concentrations (control, 40, 60, 80, 100, and 120 µg/mL). Following incubation at room temperature for 30 minutes, the resulting absorbance of the solutions was measured at 504 nm.

Toxicity assay

Zebrafish eggs were cultured with different concentrations of the bMgO NPs to assess the degree of mortality and determine the cytotoxic effects of the prepared NPs. Dilutions of bMgO NPs in different concentrations were used. Saline was used as the control. In a processing tank of Hank’s solution, 10 zebrafish eggs were introduced. Different concentrations of the bMgO NPs were added to each well containing a zebrafish egg and allowed for embryogenesis. The wells were maintained at a stable room temperature of 24°C. The development and formation of organs were observed. The optical microscope (CX41; Olympus Corporation, Tokyo, Japan) was used to observe the zebrafish embryos. The viability of the embryos was checked every eight hours, and the dead embryos were discarded to restrict contamination.

Statistical analysis

The data were collected, tabulated, and analyzed using IBM SPSS Statistics for Windows, Version 23.0 (Released 2015; IBM Corp., Armonk, NY, USA). A one-way ANOVA was performed to assess the antibacterial activity of bMgO NPs in comparison to the antibiotic standard, with a p value ≤ 0.05 set as statistically significant. An unpaired t test was performed to assess the antioxidant activity and cytotoxicity of bMgO NPs in comparison to the standard, with a p value ≤ 0.05 set as statistically significant.

## Results

Antimicrobial assay

**Figure 1:**
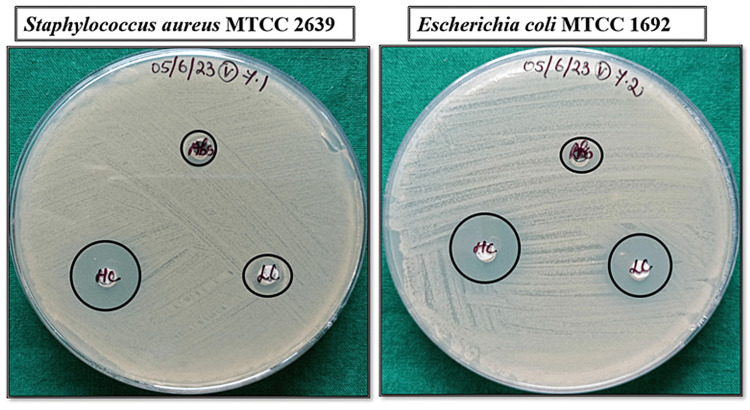
Antibacterial analysis of bMgO NPs against Staphylococcus aureus and Escherichia coli Abs: antibiotic standard; bMgO NPs: *Bacopa monnieri*-mediated synthesis of magnesium oxide nanoparticles; HC: high concentration; LC: low concentration; MTCC: Microbial Type Culture Collection and Gene Bank

The antimicrobial properties of the green synthesized bMgO NPs have been depicted in Figure 1 and Table 1. A combination of erythromycin 25 mg and amoxicillin 25 mg in a ratio of 1:1 was used as the antibiotic control. The zone of inhibition against both bacterial strains increased with the increase in the concentration of the bMgO NPs. For *S. aureus*, on comparing the zone of inhibition of the antibiotic standard with a high concentration of bMgO NPs, a statistically significant difference was noted. Similarly, for *E. coli*, on comparing the zones of inhibition of the antibiotic standard with the low concentration of bMgO NPs and on comparing the zones of inhibition of the antibiotic standard with the high concentration of bMgO NPs, a statistically significant difference was noted. Thus, the bMgO NPs have shown excellent antibacterial properties against *S. aureus* and *E. coli* compared to the antibiotic standard.

**Table 1:** Comparison of antibacterial activity of bMgO NPs and antibiotic standard - one-way ANOVA ^*^p ≤ 0.05 set as statistically significant bMgO NPs: *Bacopa monnieri*-mediated synthesis of magnesium oxide nanoparticles; MTCC: Microbial Type Culture Collection and Gene Bank

Strain	Concentration	N	Mean (zone of inhibition)	Standard deviation	P value
*Staphylococcus aureus *(MTCC 2639)	Antibiotic standard	5	10.000	1.5811	0.085
	bMgO NPs - low concentration	5	14.000	2.5495	0.486
	bMgO NPs - high concentration	5	16.000	3.5355	0.010^*^
*Escherichia coli *(MTCC 1692)	Antibiotic standard	5	12.000	2.5495	0.001^*^
	bMgO NPs - low concentration	5	18.000	1.5811	0.706
	bMgO NPs - high concentration	5	19.000	1.5811	0.000^*^

DPPH assay

**Figure 2:**
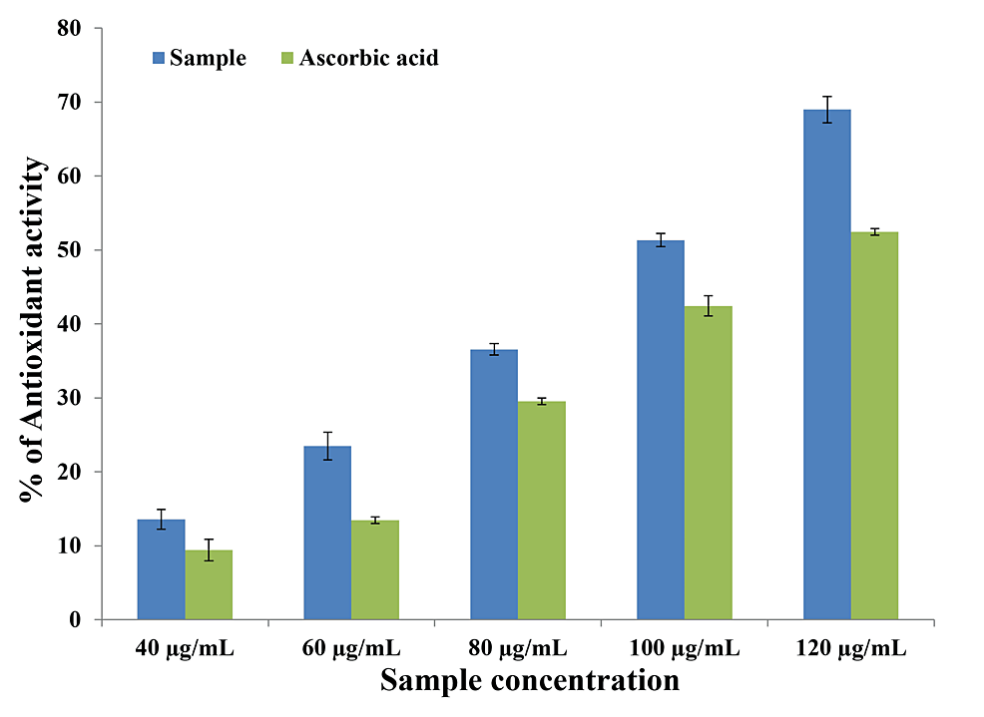
Antioxidant analysis of bMgO NPs using the DPPH assay bMgO NPs: *Bacopa monnieri*-mediated synthesis of magnesium oxide nanoparticles; DPPH, 1,1-diphenyl-2-picrylhydrazyl

The standard antioxidant activity at 40, 60, 80, 100, and 120 µg/mL was plotted for the control vitamin C, and the derived equation was used to analyze the quantity of antioxidants in the sample. The results of the DPPH assay are shown in Figure 2 and Table 2. The antioxidant activity increased in accordance with the increase in the concentration of the bMgO NPs. At all concentrations, no statistically significant difference was noted between the antioxidant activity of the bMgO NPs and the control, thus proving that the developed bMgO NPs have excellent antioxidant activity that is comparable to that of the gold standard for vitamin C.

**Table 2:** Comparison of antioxidant activity of bMgO NPs and vitamin C using the DPPH assay - unpaired t test ^*^p ≤ 0.05 set as statistically significant bMgO NPs: *Bacopa monnieri*-mediated synthesis of magnesium oxide nanoparticles; DPPH, 1,1-diphenyl-2-picrylhydrazyl

Concentration	Sample	N	Mean (% of antioxidant activity)	Standard deviation	P value
40 µg/ml	bMgO NPs	5	30.000	4.7434	0.077
	Vitamin C	5	20.000	1.5811	
60 µg/ml	bMgO NPs	5	40.000	4.4721	0.389
	Vitamin C	5	30.000	6.0827	
80 µg/ml	bMgO NPs	5	50.000	5.1478	0.389
	Vitamin C	5	40.000	7.2801	
100 µg/ml	bMgO NPs	5	60.000	3.1622	1.000
	Vitamin C	5	55.000	3.1622	
120 µg/ml	bMgO NPs	5	70.000	3.1622	0.713
	Vitamin C	5	65.000	3.5355	

H_2_O_2_ assay

**Figure 3:**
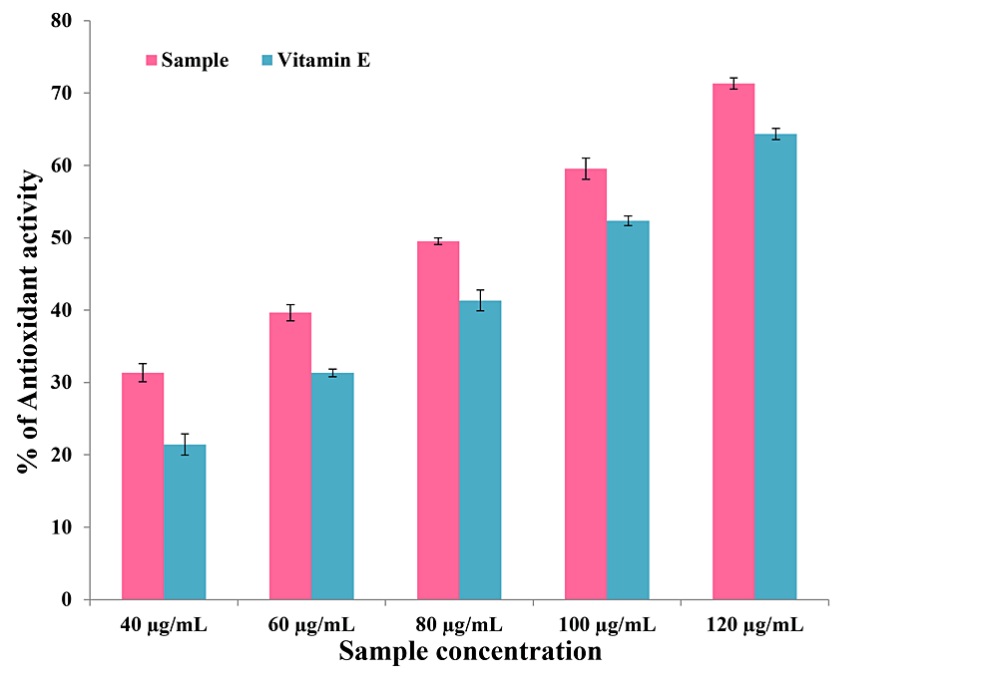
Antioxidant analysis of bMgO NPs using the H2O2 assay bMgO NPs: *Bacopa monnieri*-mediated synthesis of magnesium oxide nanoparticles; H_2_O_2_: hydrogen peroxide

The antioxidant activity was assessed using the H_2_O_2_ assay, and the results are shown in Figure 3 and Table 3. Vitamin E was used as the control. The antioxidant activity increased with the increase in the concentration of the bMgO NPs. At all concentrations, although the bMgO NPs expressed better peroxidase activity than vitamin E, no statistically significant difference was noted between the antioxidant activity of the bMgO NPs and vitamin E. The work demonstrates the great potential of bMgO NPs as antioxidants, anti-inflammatory agents, and anticancer agents.

**Table 3:** Comparison of antioxidant activity of bMgO NPs and vitamin E using the H2O2 assay - unpaired t test ^*^p ≤ 0.05 set as statistically significant bMgO NPs: *Bacopa monnieri*-mediated synthesis of magnesium oxide nanoparticles; H_2_O_2_: hydrogen peroxide

Concentration	Sample	N	Mean (% of antioxidant activity)	Standard deviation	P value
40 µg/ml	bMgO NPs	5	31.200	5.3572	0.894
	Vitamin E	5	20.000	5.3851	
60 µg/ml	bMgO NPs	5	40.000	6.0415	0.407
	Vitamin E	5	30.000	8.0622	
80 µg/ml	bMgO NPs	5	50.000	6.6708	0.514
	Vitamin E	5	40.000	8.6313	
100 µg/ml	bMgO NPs	5	60.000	6.3245	0.265
	Vitamin E	5	55.000	3.5355	
120 µg/ml	bMgO NPs	5	70.000	7.1063	1.000
	Vitamin E	5	65.000	6.0415	

Toxicity assay

**Figure 4:**
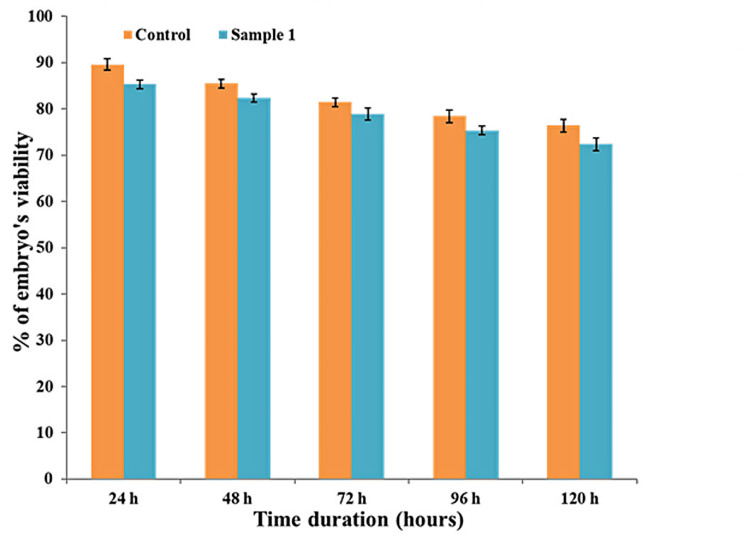
Cytotoxicity analysis of bMgO NPs observed in zebrafish bMgO NPs: *Bacopa monnieri*-mediated synthesis of magnesium oxide nanoparticles

**Figure 5:**
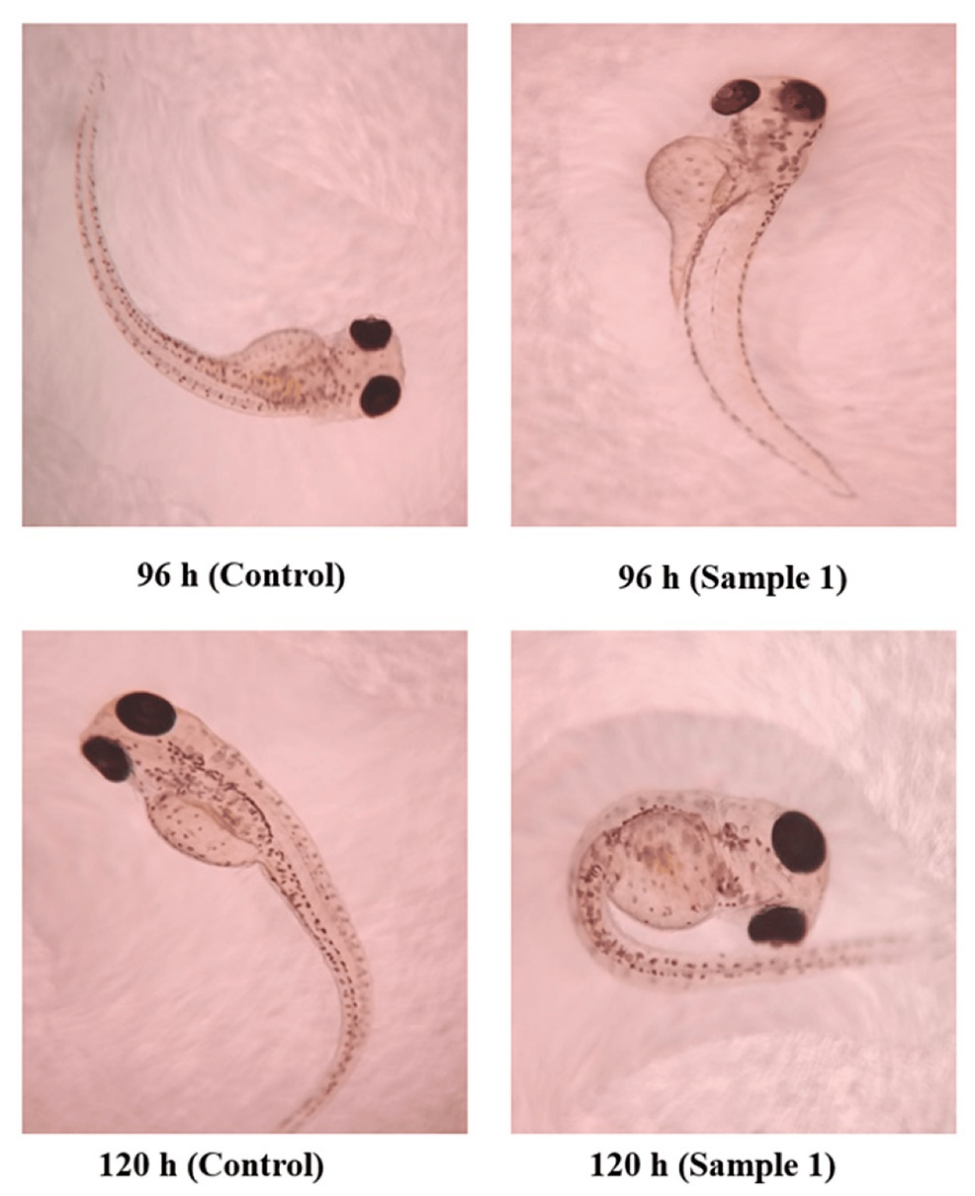
Zebrafish embryo development noted at different stages

In vitro toxicity studies were conducted using zebrafish embryos. Saline was used as the control. The toxicity was analyzed based on the viable percentage of embryos after treatment with bMgO NPs. The results are shown in Figure 4 and Table 4. The zebrafish embryos were analyzed using a light-field microscope at 40x magnification. The viability of bMgO NPs-treated embryos was comparable to that observed with the control. The bMgO NPs-treated zebrafish embryos were well formed with appropriate head, tail, eye, and internal organ development at the correct time intervals, as seen in Figure 5. At 92 and 120 hours, several embryos had hatched and displayed various growth stages, as seen in Figure 5. On comparing the cytotoxicity effect of bMgO NPs with the control on the viability of zebrafish embryos, no statistically significant difference was noted. The results indicated the bMgO NPs to be highly biocompatible with very limited toxicity to the developing zebrafish embryos.

**Table 4:** Comparison of cytotoxicity of bMgO NPs and control - unpaired t test *p ≤ 0.05 set as statistically significant bMgO NPs: *Bacopa monnieri*-mediated synthesis of magnesium oxide nanoparticles

Time	Sample	N	Mean (% of embryo viability)	Standard deviation	P value
24 hours	Control	5	90.000	7.2801	0.117
	bMgO NPs	5	85.000	3.1622	
48 hours	Control	5	85.000	4.1231	0.294
	bMgO NPs	5	83.000	2.5495	
72 hours	Control	5	82.000	3.5355	0.334
	bMgO NPs	5	80.000	6.7082	
96 hours	Control	5	79.000	4.1231	0.294
	bMgO NPs	5	77.000	2.5495	
120 hours	Control	5	78.000	2.2360	0.242
	bMgO NPs	5	75.000	3.5355	

## Discussion

With the increase in the prevalence and severity of periodontal disease, it is imperative now to find effective therapeutic modalities that aim at complete eradication of the etiologic causes leading to the diseases. Although mechanical therapy aims to achieve this, adjunctive therapeutics with antimicrobial and antioxidant properties enable us to control the established, advanced, and aggressive forms of disease more efficiently. The present study analyzed green synthesized MgO NPs using *B. monnieri *to assess their antimicrobial, antioxidant, and toxicity properties.

**Figure 6:**
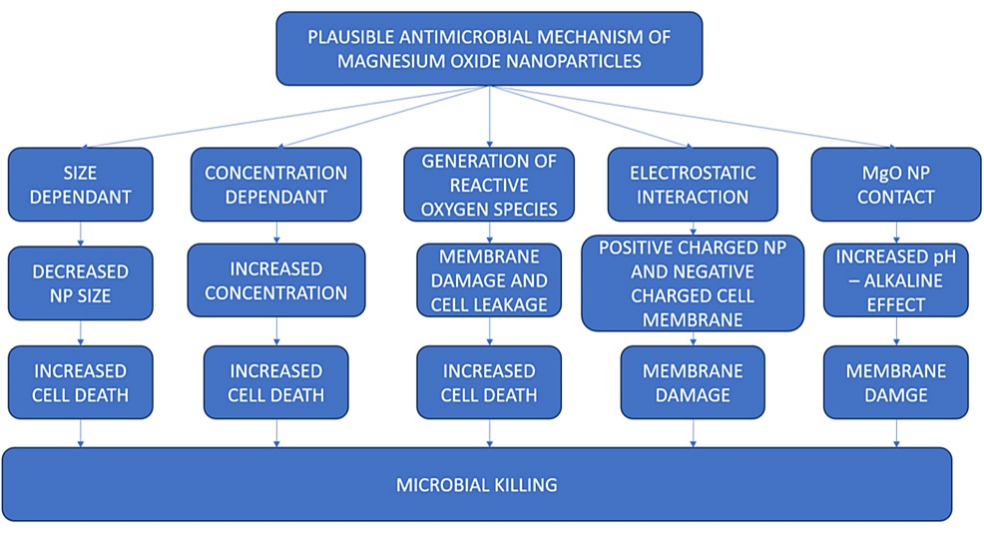
Plausible antimicrobial mechanisms of MgO NPs MgO NP: magnesium oxide nanoparticle; NP: nanoparticle

The antimicrobial assay was performed with two common oral pathogens, *S. aureus* and *E. coli*, with a combination of erythromycin and amoxicillin as the antibiotic standard. The results revealed that the green synthesized bMgO NPs had excellent antibacterial activity against both bacterial strains at low and high concentrations. The antibacterial activity of the bMgO NPs was comparatively greater than the antibiotic standard, with a larger zone of inhibition noted with bMgO NPs. Although many plausible antimicrobial mechanisms of MgO NPs have been postulated, as shown in Figure 6, the exact mechanism has not been identified yet. The antimicrobial activity of the bMgO NPs may be attributed to two different mechanisms: reactive oxygen species-mediated and non-reactive species-mediated antimicrobial action. MgO NPs have been shown to produce H_2_O_2_, leading to oxidative stress within the microbial system [10]. This leads to the production of reactive species, which leads to cell death. Also, MgO NPs have been implicated in cell membrane damage and leakage of cellular contents following physical contact [11]. Their relatively smaller size enables them to enter the cells faster, and their increased surface area allows for greater cell interactions. Higher concentrations of the MgO NPs have been proven to cause cell, protein, and DNA damage. The antibacterial assay results of this study are in accordance with other studies that have proven the antimicrobial potential of MgO NPs against both gram-positive and gram-negative bacteria [12-15].

The antioxidant activity of bMgO NPs was assessed using DPPH and H_2_O_2_ assays. Vitamin C was the control in the DPPH assay, while Vitamin E was the control in the H_2_O_2_ assay. The green synthesized bMgO NPs showed excellent antioxidant activity comparable to that observed with the control, vitamin C, and vitamin E. Similar results were noted in studies that assessed the antioxidant properties of green synthesized MgO NPs using various green extracts [16,17]. This may be attributed to the chelating property of magnesium ions, which enhances their radical scavenging ability. This magnesium-induced chelation has been reported to increase the resistance of cells to oxidative stress. The enhanced antioxidant activity of bMgO NPs may also be attributed to the high phytochemical content in the *B. monnieri* plant [9]. Phytochemical screening of *B. monnieri* has revealed the presence of tannins, terpenoids, flavonoids, alkaloids, phenols, etc. [18]. Studies have shown enhanced antimicrobial, antioxidant, anti-inflammatory, and anticancer properties owing to these phytochemicals [19,20], which increase the scope of the periodontal therapeutic application of bMgO NPs.

On toxicity analysis, it was observed that the bMgO NPs had excellent biocompatibility with minimal effects on the mortality ratio of the zebrafish. The viability of the zebrafish treated with the bMgO NPs was comparable to that observed with the control. The bMgO NPs-treated zebrafish were well formed at 24 and 48 hours, with the development of well-appreciable head, tail, eye, vertebrae, and internal organs at 72 hours. The treated zebrafish progressed to different stages of growth and development at a normal pace at 96 and 120 hours with minimal toxicity effects. This proved that the developed bMgO NPs were highly biocompatible and could be employed for further periodontal applications. The results of this study are in accordance with another study that analyzed the toxicity of green synthesized versus traditionally synthesized MgO NPs on zebrafish, which reported that green synthesized bMgO NPs had higher biocompatibility than traditionally synthesized MgO NPs concerning the notochord development and heartbeat of zebrafish [21]. Similarly, another study that evaluated the biocompatibility of silver NPs green synthesized using B. monnieri revealed no severe cytotoxic effects [22], which is in accordance with our results. Hence, it can be stated that the use of *B. monnieri* is safe for the green synthesis of NPs.

The results of this study prove that bMgO NPs have excellent antibacterial and antioxidant properties and biocompatibility. Hence, they have great potential for application as local drug delivery agents in treating periodontal infections. Formulations of bMgO NPs in the form of gels, lozenges, fibers, and microspheres can be employed in the treatment of deep periodontal pockets following thorough scaling and root planning. The antimicrobial properties of the bMgO NPs may be more effective in the control of intraepithelial periopathogens in the deep pockets. Their antioxidant property may add to the positive effect of these NPs, bringing the tissues back to a state of health. It may also reduce the need for a more invasive surgical approach, thereby reducing the morbidity associated with this disease. Moreover, the greener NPs synthesis method has proven to be more eco-friendly and effective than traditional synthesis methods. However, this study has a few limitations.

The antibacterial activity of bMgO NPs could have been assessed against the common periopathogens. The anti-biofilm and anti-inflammatory properties could have been assessed using cell line studies. Long-term animal and human clinical studies must be conducted to further explore the role of bMgO NPs in treating periodontal infections. The efficacy of the bMgO NPs in reducing periodontal pocket depth and increasing clinical attachment gain, as well as the pharmacokinetics, pharmacodynamics, release pattern, and degradation profile of the NPs, can also be assessed in future studies.

## Conclusions

The green synthesized bMgO NPs have shown excellent antibacterial and antioxidant properties and biocompatibility, increasing their scope as a local drug delivery agent in the treatment of periodontal infections. Exploring its potential application as a pharmaceutical option may provide us with a natural, safer drug with greater efficacy than currently available drugs. Further, long-term and clinical studies should be conducted to assess its implications for the management of periodontal disease.
